# Hybrid normal metal/ferromagnetic nanojunctions for domain wall tracking

**DOI:** 10.1038/s41598-017-06292-y

**Published:** 2017-07-24

**Authors:** Héctor Corte-León, Patryk Krzysteczko, Alessandra Manzin, Hans Werner Schumacher, Vladimir Antonov, Olga Kazakova

**Affiliations:** 1 National Physical Laboratory, Teddington, TW11 0LW United Kingdom; 2 Royal Holloway University of London, Egham, TW20 0EX United Kingdom; 3 Physikalisch-Technische Bundesanstalt, Braunschweig, D-38116 Germany; 4 Istituto Nazionale di Ricerca Metrologica, Torino, I-10135 Italy

## Abstract

Hybrid normal metal/ferromagnetic, gold/permalloy (Au/Py), nanojunctions are used to investigate magnetoresistance effects and track magnetization spatial distribution in L-shaped Py nanostructures. Transversal and longitudinal resistances are measured and compared for both straight and 90° corner sections of the Py nanostructure. Our results demonstrate that the absolute change in resistance is larger in the case of longitudinal measurements. However, due to the small background resistance, the relative change in the transversal resistance along the straight section is several orders of magnitude larger than the analogous longitudinal variation. These results prove that hybrid nanojunctions represent a significant improvement with respect to previously studied all-ferromagnetic crosses, as they also reduce the pinning potential at the junction and allow probing the magnetization locally. In addition, unusual metastable states with longitudinal domain walls along Py straight sections are observed. Micromagnetic simulations in combination with a magnetotransport model allow interpretation of the results and identification of the observed transitions.

## Introduction

Domain wall (DW) based nanotechnology holds the promise of better memory devices^1^, new logic circuits^2^, and manipulation or detection of magnetic beads^3–11^ (MBs), opening the possibility of integrating several laboratory functions into a single chip. The latter application is of large practical interest, since it can lead to automation and high-throughput screening (*i.e*. Lab-On-a-Chip) for biomedical uses^3–11^. However, in order to develop these applications, it is required to automate detection and manipulation of DWs. For these reasons, it is important to investigate physical phenomena such as anisotropic magnetoresistance (AMR) or planar Hall effect (PHE), occurring in nanostructures fabricated from a single magnetic material, and that allow tracking the DW position via electrical measurements, from which the magnetization spatial distribution can be inferred. For instance, in a typical Lab-On-a-Chip experiment^3^, a functionalized MB suspended in a fluid is attracted by the stray field gradient generated by a DW pinned inside a magnetic nanostructure. Once the MB is over the nanostructure, it can be manipulated by moving the DW along the nanostructure, or the MB presence can be detected by monitoring the magnetization change due to its proximity. In both cases, tracking of the magnetization can be achieved via magnetotransport measurements of the longitudinal (AMR configuration) or transversal (PHE configuration) resistance^6, 8, 12, 13^. Although effects such as giant magnetoresistance (GMR) provide a much larger signal than AMR or PHE^14^, they require the integration of several different magnetic materials to obtain a multilayer stack and hence the design of tracks to perform Lab-On-a-Chip measurements is a more complex task.

The small magnitude of the resistance change is one of the main challenges in AMR measurements, *e.g*. typically ~0.2% change in resistance in Py nanodevices^10, 13^. Because of the small magnitude of the AMR effect it is desirable to place electrical contacts close to each other as well as to the DW pinning position. Hence, the other significant challenge is to achieve a good alignment between nanostructures and electrodes during fabrication. On the other hand, a substantially larger change in resistance was observed with PHE measurements, *e.g*. the reversal of the magnetization in a Py nanowire of similar dimensions produces a change in resistance of ~10 mΩ over a theoretical zero resistance background^11^. However, the introduction of ferromagnetic crosses in the device geometry can create undesired pinning sites^15^, causing an increase in the switching field.

Since DW-based nanotechnology requires manipulation of a DW along a nanostructure, the limitation of pinning sites is a major requirement. This explains why longitudinal AMR measurements have been widely used to study magnetization in nanowires^6, 10, 13, 16–22^, while there are fewer studies on the transversal PHE at the nanoscale^11, 15, 23^.

Here, in order to overcome the problem of adding extra pinning sites and with the aim of exploiting the large changes in resistance reported for PHE, we present an alternative approach to PHE measurements, which consists in transversal resistance measurements using a hybrid normal metal (Au)/ferromagnetic (Py) nanojunction.

**Figure 1:**
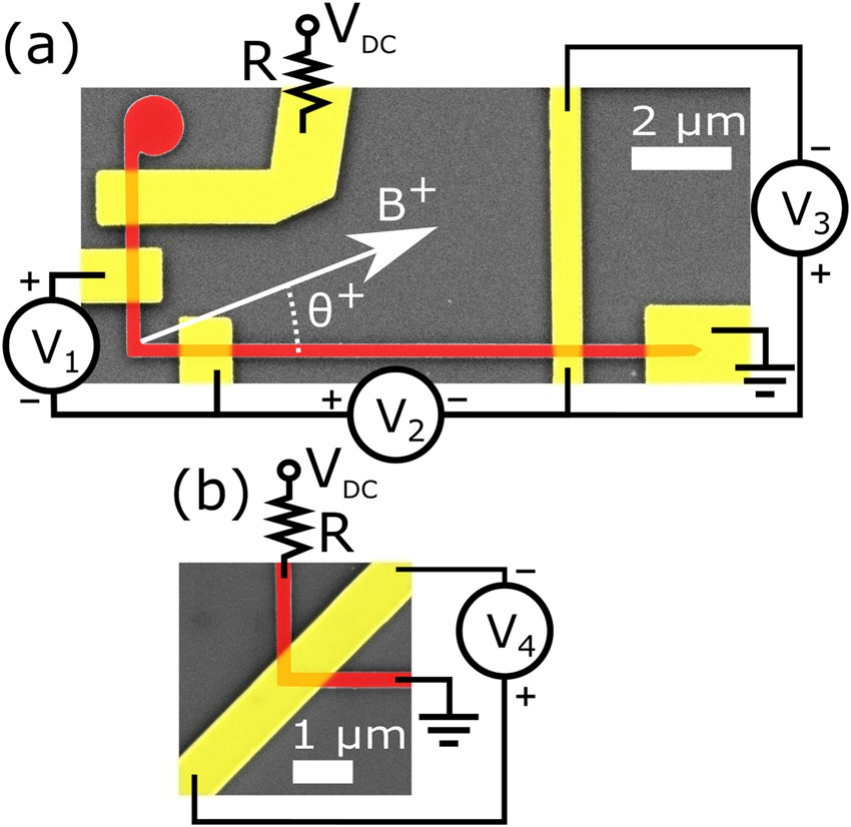
SEM images of Py/Au hybrid nanojunctions (red/yellow, respectively) using combined straight/corner (**a**) and corner only (**b**) geometries. Hybrid nanojunctions are formed of an L-shaped Py device and Au electrodes. References to the applied field and electrical circuit are shown.

Hybrid Py/Au L-shaped nanostructures (Fig. 1) with a 200 nm nominal width of Py wires are used to perform longitudinal AMR and transversal resistance measurements. We demonstrate that the L-shape devices of a given geometry can have either a DW trapped at the corner or, in a more unusual case (not stable without external field), a longitudinal DW extended along one of the straight arms. AMR and PHE measurements have been performed both across the corner and along one of the straight arms in order to compare longitudinal and transversal resistances as well as to investigate the effect of the DW presence. Our results show that the relative change in transversal resistance in the straight arm is several orders of magnitude larger than the analogous longitudinal one (*i.e*. when comparing to the value of resistance immediately before the reversal). The angular dependence of all magnetoresistive phenomena is also discussed, and interpreted with the help of *in situ* MFM imaging and micromagnetic modelling in order to elucidate the complex magnetization configuration and confirm that the proposed hybrid junctions do not introduce any additional pinning sites in the nanostructure.

## Results

**Figure 2:**
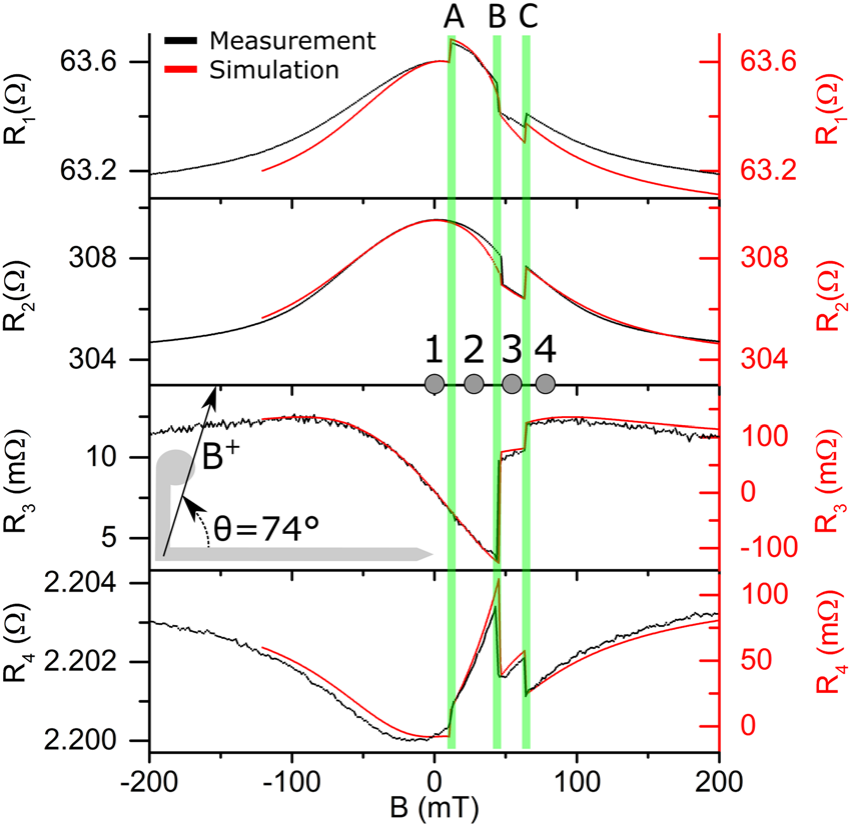
Magnetoresistance versus applied field for different measurement geometries: black - experimental and red – micromagnetic modelling results. Magnetic field is applied at *θ* = 74°, ramping from negative to positive saturation values. Longitudinal resistance along device corner (*R*_1_) and straight wire (*R*_2_), hybrid junction resistance across a straight wire (*R*_3_), and hybrid junction resistance across device corner (*R*_4_) (see Fig. 1 for electrical set-up).

**Figure 3:**
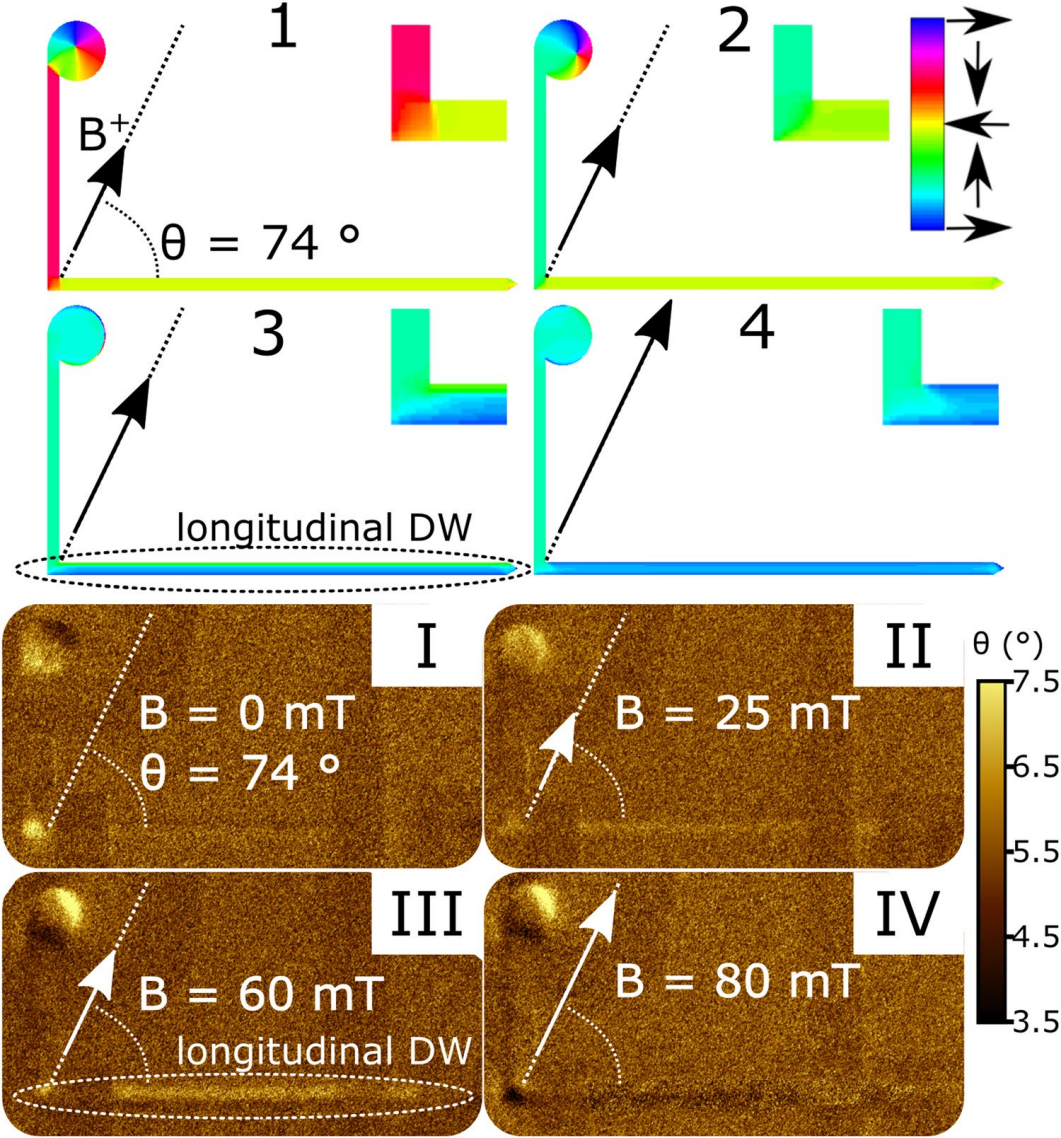
Top: Simulated magnetization spatial distribution in the L-shaped Py nanostructure at equilibrium, the magnetic field is applied at *θ* = 74°. Color scale refers to the magnetization direction with respect to the *x*-axis. Numbers (1–4) correspond to the identically labelled areas in Fig. 2. Insets show magnified images of the corner. Bottom: *in situ* MFM images (I–IV) obtained in the conditions identical to (1–4) simulations.

**Figure 4:**
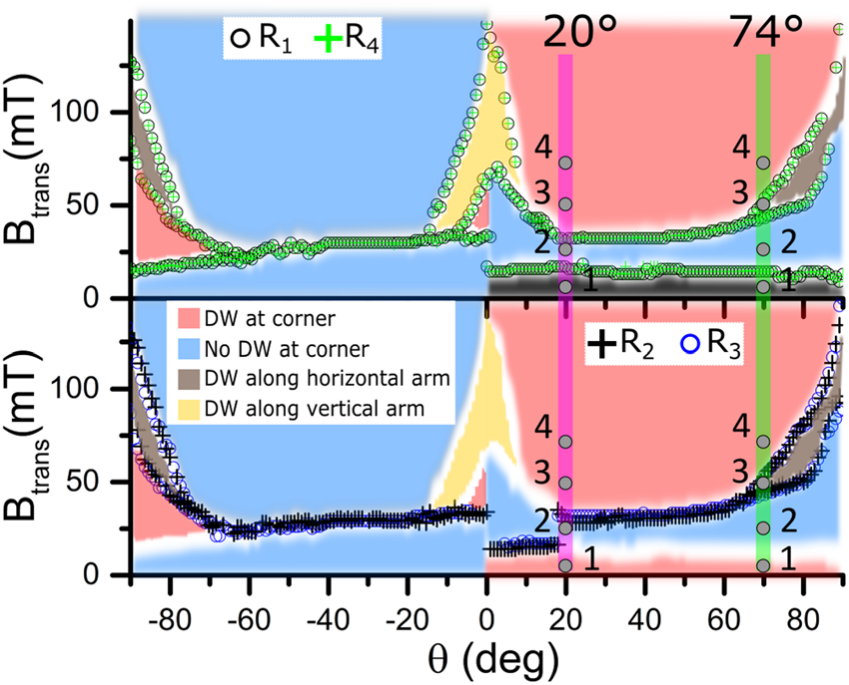
Angular dependence of irreversible transition fields and main magnetization states extracted from magnetotransport, MFM measurements, and simulations (used to infer the magnetization spatial distribution), for −90° < *θ* < 90°: top panel – resistances *R*_1_ and *R*_4_ measured at the corner; bottom panel – resistances *R*_2_ and *R*_3_ measured along the straight nanostructure. The colors correspond to the main magnetization states according to the legend. Note that the magnetization states are characteristic for the given magnetic nanostructure, whereas a specific resistance measurement serves as a probe for their determination. White bands correspond to transitions between states, which cannot be probed in specific resistance geometries. Numbers 1–4 (grey circles) on the green line at *θ* = 74° correspond to the areas described in Figs 2 and 3, numbers 1–4 on the purple line at *θ* = 20° correspond to energy evolution described in Fig. 5.

In order to analyze the different signals produced in transversal and longitudinal resistance measurements, the resistances for different types of configurations (both measured and simulated) are compared when the external magnetic field is ramped up from a negative saturating value at different angular orientations, −90° < *θ* < 90°. Measurements and simulation results shown in Fig. 2 refer to the case of *θ* = 74°, which corresponds to the formation of a longitudinal DW along the wire (to be discussed in detail in Fig. 4). The experimental set-up scheme of resistance measurements (*R*_1_-*R*_4_) corresponds to the positions of voltmeters (*V*_1_-*V*_4_) in Fig. 1. In addition to the measured and simulated resistance curves (Fig. 2), calculated magnetization maps and experimental *in situ* MFM images at *θ* = 74° are shown in Fig. 3 at key values of the magnetic field.

As magnetic field ramps from negative to positive saturation values, four characteristic areas, labelled 1–4 (see grey circular markers on the top applied field-axis), can be distinguished in Fig. 2.

Area 1 (*B* < 11 mT): first, saturating negative field is applied and then reduced to *B* = 0. The relevant modelled and experimentally measured (*i.e*. MFM) remanent magnetization states are shown in Fig. 3 (state 1 and I, respectively). The state contains a DW trapped at the corner, with magnetization uniformly distributed along the arms and vortex configuration at the nucleation disk. At *B* = 0, a zero value for transversal resistances *R*_3_ and *R*_4_ is expected as also confirmed by micromagnetic simulations, which directly model the PHE in the ferromagnetic material. However, both experimentally obtained *R*_3_ and *R*_4_ have a small but finite value, associated with current circulating through the metal electrode. It is also interesting to note that the transversal resistance *R*_3_ (*i.e*. associated with the hybrid junction over the straight part of the nanostructure) has an approximately linear field dependence (~0.1 Ω/T) in the field range −30 mT < *B* < 30 mT (Fig. 2). This result makes this type of junctions a good candidate for measurements of local magnetic fields, for example as the ones produced by magnetic nanoparticles^11, 24, 25^, without the need of additional fabrication steps (*e.g*. as required to produce a magnetic tunnel junction or a GMR based sensor composed of a multilayer stack).

Area 2 (11 mT < *B* < 45 mT): when the magnetic field is increased, the DW at the corner is annihilated and the magnetization in the vertical arm is reversed, as a consequence of the irreversible motion towards corner of the DW previously pinned at the disk-vertical arm junction, occurring at 11 mT (transition **A** in Fig. 2). Successively, magnetization spatial distribution in both arms gradually changes without re-formation of a DW at the corner, as depicted by states 2 and II in Fig. 3. At transition **A**, signals *R*_1_ and *R*_4_, which directly probe the corner state, show a step indicating a change in the resistance due to the annihilation of the DW pinned at the corner (Fig. 2). On the contrary, as the DW disappears due to the reversal of the magnetization in the vertical arm, signals *R*_2_ and *R*_3_ do not show an abrupt change at **A**.

Area 3 (45 mT < *B* < 64 mT): by further increasing the magnetic field, at 45 mT the magnetization reaches a rather counterintuitive state with a longitudinal DW along the horizontal arm (Fig. 3, states 3 and III). The associated change in the resistance (transition **B** at *B* = 45 mT) can be measured in all the configurations, as changes in the magnetization distribution occur beneath all the contacts. Also *i* *n situ* MFM images (Fig. 3 state III) demonstrate the existence of this state with a longitudinal DW along the horizontal arm^26^, which for the L-shaped nanostructure used here is not stable and thus cannot be imaged at remanence. Although this state is metastable, it is highly reproducible and always appears in a well-defined space state, *i.e*. combination of the field magnitude and orientation (see grey and yellow areas in Fig. 4), and can be possibly exploited in spin-wave propagation studies^27^.

Area 4 (*B* > 64 mT): when the magnetic field is increased even more, the longitudinal DW annihilates at 64 mT moving orthogonally to the naowire axis (transition **C** in Fig. 2), while a new DW is formed at the corner accompanied by uniform magnetization distribution along both arms (states 4 and IV in Fig. 3). This transition is reflected by steps in the resistances as measured in all the configurations *R*_1_-*R*_4_.

As follows from Fig. 2, the absolute change in the transversal resistance in *R*_3_ geometry is smaller than the analogous longitudinal measured ones (*R*_1_ and *R*_2_), however, when the change in resistance is compared with the resistance before the transition, the relative change in *R*_3_ is several orders of magnitude bigger than the changes in *R*_1_ and *R*_2_. On the other hand, the measured transversal resistance at the corner, *i.e*. for *R*_4_ geometry, is characterized by even smaller values than analogous longitudinal resistance (*R*_1_). Thus, while there is no significant difference in terms of DW tracking as measured at the corner (*i.e*. *R*_1_ or *R*_4_ for transversal or longitudinal measurements, respectively), there is a massive difference between longitudinal and transversal measurements in the straight configuration. It is noteworthy that, when compared to the simulated values reported in Fig. 2, the transversal measurements show a finite background resistance in both *R*_3_ and *R*_4_ geometries, while the simulations predict nearly zero resistance at *B* = 0. The reason for this discrepancy is associated with a significant amount of current passing through the Au contact instead of the Py nanowire at the nanojunction^28^, (see typical experimental values in the Supplementary Information, Fig. S2). This is not taken into account in the numerical model, which simulates only the PHE in the Py material. The resulting background resistance reduces the relative change for the experimental transversal measurements, thus it is expected that with a more resistive material, instead of Au, relative resistance changes in *R*_3_, and in particular in *R*_4_, could be significantly larger than the values reported in Fig. 2 (Suplementary Information, Table S1).

The combined results presented in Figs 2 and 3 demonstrate that for *θ* = 74° all the irreversible transitions observed in the experimental curves in Fig. 2 can be fully interpreted by changes in the spatial distribution of magnetization as predicted by the simulations (Fig. 3), which signifies absence of any additional pinning sites due to the presence of the hybrid junction. However, in order to fully characterize the hybrid nanojunctions and corroborate the predictions made by micromagnetic simulations, the angular dependence of the DW pinning/depinning fields (*i.e*. transitions **A**, **B**, and **C** in resistances *R*_1_–*R*_4_) was studied (results can be seen in Fig. 4). In this case, the resistances for each angular orientation were measured twice and the average field of the transition is plotted (transitions are considered the same if their field separation is less than 0.5 mT).

Figure 4 top panel demonstrates that measurements across the corner (*R*_1_ and *R*_4_) show similar angular transitions occurring at the same fields. Correspondently, measurements of the straight nanostructure (*R*_2_ and *R*_3_) also provide the same angular transitions (Fig. 4 bottom panel), though different from the set of measurements at the corner. This demonstrates the absence of any additional DW pinning sites induced by the electrical contacts in the whole field angular range (*e.g*. a DW pinned at the hybrid junction position would appear as an extra transition in *R*_3_ or *R*_2_).

Figure 4 also shows the different magnetization states that appear in the nanostructure when the field is ramped at different angles (states are shown by different colors, pink, blue, grey and yellow), classified by analyzing the sign of the resistance change in measurements *R*_1_-*R*_4_, MFM images at specific angles, and simulation results. It should be noted that these 4 possible states are characteristic for the given magnetic nanostructure, *i.e*. they exist independently of the way of measurements. Depending on the orientation of the applied field, either 2 or 3 transitions can be observed, as for example shown for *θ* = 20° and 74°, respectively. In particular, when *θ* = 20° the magnetization reversal occurs again first in the vertical nanowire and second in the horizontal one, but in this case the switching mechanism in the horizontal nanowire is no more characterized by two steps, with the formation of the longitudinal DW.

By analysing Fig. 4, it is possible to define the state space conditions favourable for the generation of the metastable states with a longitudinal DW along the horizontal/vertical arm^29^, as depicted by the grey/yellow color in Fig. 4 (*i.e.* as shown in Fig. 3 state III for a horizontal DW). In particular, Fig. 4 shows that metastable states with a longitudinal DW along one of the arms can only be observed when the magnetic field ramps, at a fixed angular orientation, from a negative saturation field value to the field values shown in Fig. 4 with colors grey and yellow, being −90° ≤ *θ* ≤ −80° or 70° ≤ *θ* ≤ 90° for DW along the horizontal arm (grey), and −15° ≤ *θ* ≤ 15° for DW along the vertical arm (yellow). For this reason, *θ* = 74° was chosen as a characteristic angle allowing the observation of unusual magnetic states both in resistance (Fig. 2) and MFM measurements (Fig. 3 bottom).

**Figure 5:**
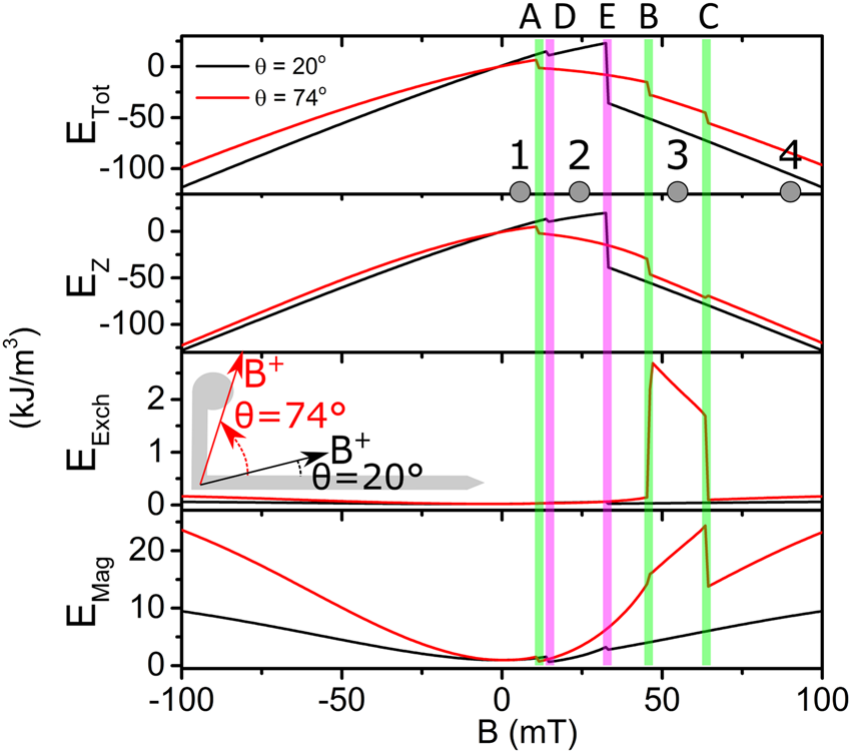
Calculated energy density contributions versus applied field for two angular orientations of the applied field, *θ* = 20° and 74°. Note different scales for individual energy terms. Even if the entire nanostructure is micromagnetically simulated, energies have been estimated eliminating the shape energy contributions due to the arm ends.

We further analyze the transitions and states between them at angular orientation *θ* = 20°. At this angle no longitudinal DW formation is observed (*i.e*. contrary to *θ* = 74°). The calculated energy density evolution versus the applied field is shown in Fig. 5 for *θ* = 20° and 74° (black and red lines, respectively). Steep decrease in the total energy *E*_tot_ occurs when the system undergoes one of the transitions: **A**, **B**, or **C** for *θ* = 74° (green vertical line); **D** and **E** for *θ* = 20° (pink vertical line). The presence of the longitudinal DW at *θ* = 74°, between transitions **B** and **C**, contributes to the overall reduction in the total energy. Considering the individual energy terms, presence of the longitudinal DW in this field range unavoidably results in an increase in the exchange, *E*_Exch_, and magnetostatic, *E*_Mag_, energies. However, it is overcompensated by the consequent reduction of the Zeeman energy, *E*_Z_, note the significantly different scales for individual energy terms in Fig. 5. Since the Zeeman energy depends on the external field, the longitudinal DW is stable only in field and does not exist at remanence. Moreover, this type of magnetization configuration appears only when the applied field has a predominant component along the direction orthogonal to the involved nanostructure arm. A stronger minimization of *E*_Z_ would require complete alignment of the magnetization with the applied field (*i.e*. perpendicular to the arm), but this would imply a great increase in the magnetostatic energy, *E*_Mag_. The formation of the longitudinal DW corresponds to minimization of the total energy *E*_tot_ and represents a good compromise between a moderate reduction in *E*_Z_, and a limited increment of *E*_Mag_ (whereas *E*_Exch_ provides a less important contribution).

For *θ* = 20°, the evolution from **D** to **E** leads to an increase the total energy, with an increment of all the three terms. At transition **E** the system evolves reducing the total energy with the generation of a DW at the corner. In this case, the formation of a longitudinal DW is not possible, since it would imply a strong increase in *E*_Z_.

## Conclusions

By combining longitudinal (AMR) and transversal (PHE) resistance and *in situ* MFM measurements with micromagnetic simulations in Py/Au L-shaped nanostructures, we demonstrate the possibility to track the magnetization state through the use of hybrid normal metal/ferromagnetic nanojunctions.

Longitudinal and transversal resistance measurements at the nanostructure corner and along one of the straight arms have been compared for different types of transitions in the magnetization spatial distribution, by varying the amplitude and angular orientation of the applied field with respect to the nanostructure. For the corner, the comparison reveals a larger absolute resistance change in the case of longitudinal measurements (*i.e*. change in resistance with respect to resistance before the transition). However, based on micromagnetic simulations it is expected that the transversal resistance change, while smaller than longitudinal one in absolute terms, could be significantly larger as a relative value, *i.e*. in relation to the resistance before the transition, if a highly resistive material is used for electrical connections (*e.g*. Ta or Ti). On the other hand, in the straight nanostructure geometry, the transverse measurements always show a significantly larger change in the relative resistance (*i.e*. >100 times). Thus, transversal resistance measurements hold the promise of much larger differences between different magnetization states, improving DW tracking and facilitating the sensing procedure.

In terms of equilibrium magnetization states, we have demonstrated that in the majority of cases the same information can be accessed either from longitudinal or from transversal measurements. In particular, the results obtained from the experimental measurements and the micromagnetic simulations have proved that the hybrid junctions do not add new pinning sites for the DWs. In addition, the measurements have allowed to identify a rare metastable state with a longitudinal DW extending along the length of the straight Py nanowire. The existence of such unusual metastable state is further proven by means of *in situ* MFM and energy considerations from micromagnetic simulations.

As a general conclusion, these results demonstrate the possibility of performing transversal measurements in submicron nanowires without adding ferromagnetic crosses and hence with less pinning sites for DW movement. In addition, since alignment during the fabrication process is less critical in the case of a single hybrid junction, this technique represents an improvement in terms of fabrication and DW detection in nanostructures.

## Methods

The magnetic L-shaped nanodevices with width of 200 nm were fabricated from a continuous polycrystalline Py/Pt film (25/2 nm) on top of a Si/SiO_x_ substrate. The main design comprises two arms: one of 10 µm in length with a taped end; another one 4 µm long and with a 1-µm disk at the end (Fig. 1a). Electrical contacts were prepared by sputtering deposition of Ta/Au (6/150 nm). Two varieties of the main design were studied. In the first case, two pairs of Ta/Au leads were positioned on both sides of the L-shape corner (Fig. 1a). In the second design, a single Ta/Au lead was fabricated directly on top of the Py corner (Fig. 1b). AFM profiles of the fabricated devices can be seen in the Supplementary Information (Fig. S1).

Figure 1 shows the schematic representation of the used electrical circuits. Resistance values *R*_1_-*R*_4_ were extracted from measured voltages, labelled *V*_1_-*V*_4_ in Fig. 1a and b, while a DC current (typical value of 100 µA) was applied. To current bias the device, a DC voltage *V*_DC_ is applied through a resistor, *R*~100 kΩ, in series with the nanostructure. The large value of this resistor, when compared with the resistance of the nanostructure (*R*~1 kΩ including the electrical connections) and with the maximum magnetoresistance change, ~ ± 5 Ω, fixes the amount of current flowing in the circuit despite of the magnetoresistive effects.

External in-plane magnetic field is applied using an electromagnet (see Fig. 1a for the angular reference), ramped at 3 mT/s. The device is placed between the poles of the electromagnet using a rotating stage that allows its precise orientation with respect to the field direction. Alignment is made by extracting the DW pinning/depinning fields in the range −90° ≤ *θ* ≤ 90° in *V*_1_ geometry (*θ* is the angle between the applied field direction and the longer arm). The maximum difference between DW pinning/depinning fields occurs when the magnetic field is parallel to one of the arms of the L-shaped nanostructure^13^.

*In situ* MFM images were taken using a scanning probe microscopy system that allows applying in-plane magnetic field during scanning (Aura, NT-MDT). In order to minimize the interaction between the sample and magnetic probe, the topography was measured only once at the beginning of the experiment, then the magnetic signal was recorded at 80 nm lift height and after applying different external magnetic fields.

The simulations were performed by means of a micromagnetic - magnetotransport numerical model able to describe both AMR and PHE phenomena. At each equilibrium point, the spatial distribution of the magnetization is computed by using a parallelized micromagnetic solver^30–32^ based on the integration of the Landau-Lifshitz-Gilbert equation. Then, the electric potential ϕ is calculated by solving the following equation:

1 $$\nabla \cdot [\overleftrightarrow{\sigma }({\bf{r}})\nabla {\rm{\varphi }}({\bf{r}})]=0$$

where the spatially dependent conductivity tensor $\overleftrightarrow{\sigma }({\bf{r}})$ is expressed as

2 $$\overleftrightarrow{\sigma }({\bf{r}})=\frac{1}{{\rho }_{\parallel }{\rho }_{\perp }}[\begin{array}{cc}{\rho }_{\parallel }-{\rm{\Delta }}\rho \,{\cos }^{2}\eta ({\bf{r}}) & -\frac{1}{2}{\rm{\Delta }}\rho \,\sin \,2\eta ({\bf{r}})\\ -\frac{1}{2}{\rm{\Delta }}\rho \,\sin \,2\eta ({\bf{r}}) & {\rho }_{\parallel }-{\rm{\Delta }}\rho \,{\sin }^{2}\eta ({\bf{r}})\end{array}],$$

In equation (2) ${\rm{\Delta }}\rho =\,{\rho }_{\parallel }-{\rho }_{\perp }$, with *ρ*_||_ and ${\rho }_{\perp }$ being the electrical resistivities measured parallel and perpendicular to the magnetization direction respectively, oriented at an angle *η*(**r**) with respect to *x*-axis^33–35^.

Equation (1), defined in a domain corresponding to the only magnetic nanostructure, is coupled to boundary conditions on current contacts and insulating boundaries, and it is iteratively solved until convergence of electrical conductivity^36^.

Here, the saturation magnetization of Py is fixed to 860 kA/m, the exchange constant to 13 pJ/m, and the magnetocrystalline anisotropy is considered negligible. Magnetotransport parameters are adopted from^37^: ${\rho }_{\parallel }=0.340\,{\rm{\mu }}{\rm{\Omega }}m$ and ${\rho }_{\perp }=0.333\,{\rm{\mu }}{\rm{\Omega }}m$.

## Electronic supplementary material

Supplementary Information
