# Supplementary material for: Hybrid normal metal/ferromagnetic nanojunctions for domain wall tracking
Source: Sci Rep. 2017 Jul 24;7:6295. doi: 10.1038/s41598-017-06292-y (PMC5524695; doi:10.1038/s41598-017-06292-y)
Supplement: Supplementary file 1 — Supplementary Information [file 41598_2017_6292_MOESM1_ESM.doc]

Hybrid normal metal/ferromagnetic nanojunctions for domain wall tracking – Supplementary Information

Héctor Corte-León^1,2^, Patryk Krzysteczko^3^, Alessandra Manzin^4^, Hans Werner Schumacher^3^, Vladimir Antonov^2^, and Olga Kazakova^1^

*^1^National Physical Laboratory, Teddington, TW11 0LW, United Kingdom*

*e-mail address:* [*hector.corte@npl.co.uk*](mailto:hector.corte@npl.co.uk)

*^2^Royal Holloway University of London, Egham, TW20 0EX, United Kingdom*

*^3^Physikalisch-Technische Bundesanstalt, Braunschweig, D-38116, Germany*

*^4^Istituto Nazionale di Ricerca Metrologica, Torino, I-10135, Italy*

1. Cross sections

Figure S1 includes transversal profiles of the devices presented here. The height profiles were taken using AFM in the non-contact mode and they were used to provide geometrical parameters for the numerical simulations.


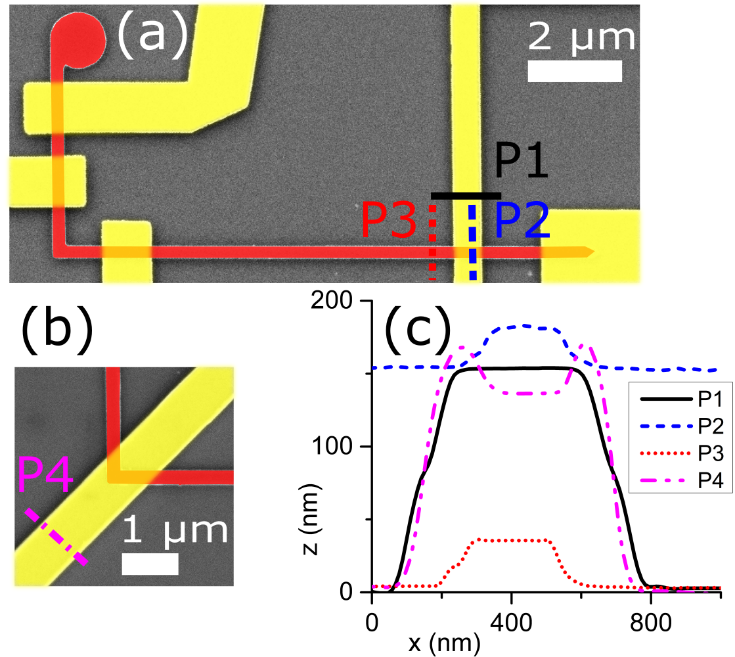


FIG. S1. (Color online) SEM images of Py/Au hybrid nanojunctions (red/yellow respectively) for the straight/corner (a) and corner only (b) electrical circuit geometries. (c) Height profiles of transversal sections indicated in (a) and (b).

1. Different widths of devices

To complement the results relative to the hybrid junction located along the nanostructure arm (R_3_), nanostructures with different widths of Py and Au layers were fabricated and tested. Figure S2a shows the change in resistance R_3_ at transition **B** for field angular orientation θ = 74º. It is important to notice that the magnetic field required to achieve transition **B** depends on the width of the Py nanostructures. Fig. S2b shows the measured values of transversal resistance R_3_ at zero field (remanence) for the same angle.


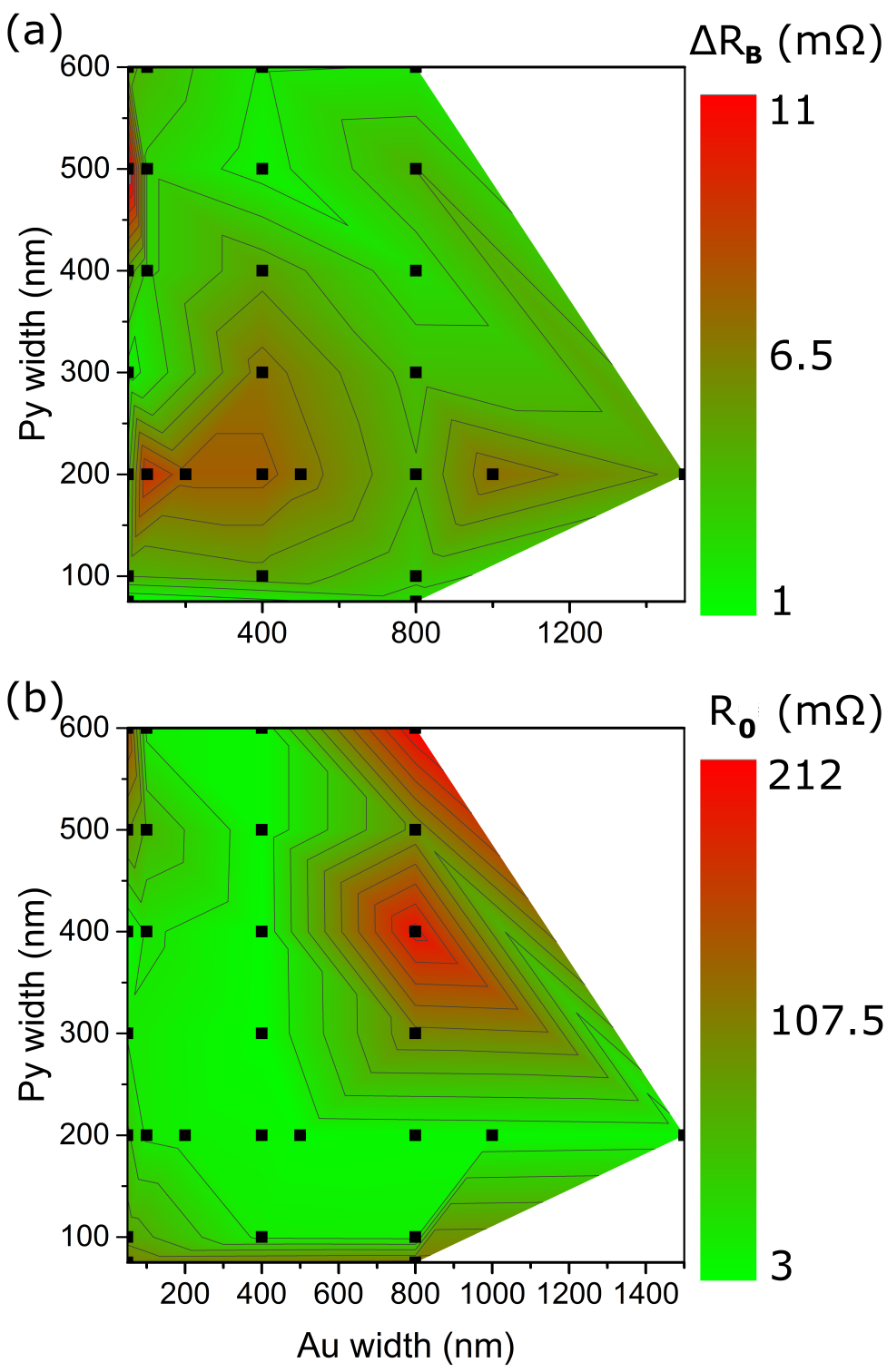


FIG. S2. (Color online) (a) Average change in the resistance R_3_ at transition **B** for θ = 74º considering different widths of Py and Au layers. (b) Average resistance R_3_ at B=0 for the same widths as in (a).

The black dots in Fig. S2 represent the tested widths (average measured values for 2-6 devices), the color map is an interpolation of the measured values. The change in the resistance associated with transition **B** is in the range 1-11 mΩ. For the device studied in the paper the change in resistance is ~6 mΩ. Hence, the reported results can be considered representative of this type of hybrid junctions. In terms of resistance at zero field (Fig. S2b) the tested device has a resistance R_3_ of ~5 mΩ. This is a low value when compared with all the tested devices analyzed in Fig. S2b, but for devices of similar widths it is close to the average resistance at zero field.

1. Absolute and relative RESISTANCE VALUES

From the experimental curves in Fig. 2, the resistance values and changes in resistance at transitions **A**, **B**, and **C** have been deduced and summarized in Table S1.

Table S1. Absolute and relative resistance at transitions **A**, **B** and **C** as extracted from Fig. 2. Black font – measured data, red font – simulated values.

| Measured  Simulated | **R before transition A (Ω)** | ΔR **at transition A** **(mΩ)** | **R before transition B**  **(Ω)** | ΔR **at transition B** **(mΩ)** | **R before transition C**  **(Ω)** | ΔR **at transition C** **(mΩ)** |
| --- | --- | --- | --- | --- | --- | --- |
| Configuration R_1_ | 63.6  63.6 | 59.9  85.0 | 63.5  63.5 | -104.0  -85.0 | 63.4  63.3 | 48.0  71.0 |
| Configuration R_2_ | 309.5  309.4 | --  -- | 308.1  307.38 | -1093  -419 | 306.4  306.2 | 1254  1382 |
| Configuration R_3_ | 0.01  -0.029 | --  -- | 0.00  -0.13 | 6.17  200.03 | 0.01  0.08 | 1.50  43.81 |
| Configuration R_4_ | 2.20  -0.00 | 0.32  0.02 | 2.20  0.00 | -1.78  -0.07 | 2.20  0.00 | -0.96  -0.03 |
